# Autophosphorylation of Orphan Receptor ERBB2 Can Be Induced by Extracellular Treatment with Mildly Alkaline Media

**DOI:** 10.3390/ijms20061515

**Published:** 2019-03-26

**Authors:** Oxana V. Serova, Natalia A. Chachina, Elena A. Gantsova, Nadezhda V. Popova, Alexander G. Petrenko, Igor E. Deyev

**Affiliations:** Shemyakin–Ovchinnikov Institute of Bioorganic Chemistry, Russian Academy of Sciences, Moscow 117997, Russia; oxana.serova@gmail.com (O.V.S.); n_chachina@inbox.ru (N.A.C.); gantsova@mail.ru (E.A.G.); npopova@gmail.com (N.V.P.); petrenkoag@gmail.com (A.G.P.)

**Keywords:** receptor tyrosine kinase, tyrosine phosphorylation, alkaline medium, pH sensor

## Abstract

ErbB2 is an oncogene receptor tyrosine kinase linked to breast cancer. It is a member of the epidermal growth factor receptor (EGFR) minifamily. ErbB2 is currently viewed as an orphan receptor since, by itself, it does not bind EGF-like ligands and can be activated only when overexpressed in malignant cells or complexed with ErbB3, another member of the EGFR minifamily. Here, we report that ErbB2 can be activated by extracellular application of mildly alkaline (pH 8–9) media to ErbB2-transfected cells. We also show that the activation of the ErbB2 receptor by alkali is dose-dependent and buffer-independent. The endogenous ErbB2 receptor of A431 cell line can also undergo alkali-dependent autophosphorylation. Thus, we describe a novel ligand-independent mechanism of ErbB2 receptor activation.

## 1. Introduction

The EGFR minifamily, one of the most intriguing of the receptor tyrosine kinase groups, consists of four members named ErbB1–4 [1]. These highly homologous receptors consist of a glycosylated extracellular ligand-binding domain, one hydrophobic transmembrane segment, and the intracellular portion that contains a tyrosine kinase domain [2]. The first discovered ligand for the ErbB receptors was epidermal growth factor (EGF). Currently, seven ligands are known to bind to ErbB1/EGFR, two ligands bind to ErbB3, and seven ligands bind to ErbB4 [2]. The interaction of ligands with ErbB receptors is not strictly selective. For example, EGF preferentially binds to the EGFR receptor, while neuregulins (NRGs), bind to both ErbB3 and ErbB4 [3].

ErbB receptors are expressed as monomers but function as homo- and heterodimers, formed upon ligand binding [2]. After the extracellular ligand binding and receptor dimerization, the kinase intracellular domains form an asymmetric complex and phosphorylate each other [2]. Two members of the ErbB family deviate from this mechanism typical for most receptor tyrosine kinases. As shown by structural studies, ErbB2 is unable to bind a ligand because its extracellular ligand-binding domain is already in a ligand-related conformation, thereby blocking the access of any other peptide ligand to this region [2]. The second “irregular” member is ErbB3, in which the kinase activity of the intracellular domain is corrupted due to a mutation in several conservative residues within the catalytic domain. It is currently believed that ErbB2 and ErbB3 are not fully fledged receptors, and for their activation, they must form a heterodimer complex which, after binding a ligand (for example, neuregulin), is autophosphorylated, triggering intracellular signaling [4]. It is currently believed that ErbB2 activation occurs only either at non-physiologically high levels of expression (e.g., in cancer cells), which leads to ligand-independent receptor phosphorylation, or as a part of the ErbB3 heterodimers complexed with neuregulin [4]. ErbB2 activation was observed in bladder, lung, gastric, ovarian, prostate, and breast cancer cells. This phenomenon is attributed to amplification of the *ErbB2* gene that results in the overexpression of ErbB2 protein at the cell surface [5]. The amplification of the *ErbB2* gene is associated with more aggressive disease, poor prognosis, and decreased overall survival [6].

Earlier, we have found that orphan insulin receptor-related receptor (IRR), a receptor tyrosine kinase of the insulin receptor minifamily, can be activated by alkaline media at pH >7.9 [7,8]. We conducted a bioinformatic search for other tyrosine kinase receptors that may have similar properties, and unexpectedly found that orphan ErbB2 receptors can also undergo pH-dependent autophosphorylation.

## 2. Results and Discussion

Our recent finding that IRR, an insulin receptor structural homolog, is a pH sensing receptor and a regulator of the acid–base balance prompted a search for other receptor tyrosine kinases that are regulated by pH changes. We hypothesized that the bioinformatic program AcalPred [9,10]—that had been originally developed for predicting media pH values, either acidic or alkaline, that are optimal for enzyme activity merely on the basis of their amino acid sequences—can be applied to predict the pH sensitivity of proteins other than enzymes. Upon input of an amino acid sequence, AcalPred calculates the relative probability that the protein “prefers” to function either in an alkaline or acidic medium, with their total being 1. Although this algorithm was originally developed and validated for soluble enzymes, we applied it for sequences of the ectodomains of the insulin receptor family from about 20 species (from frog to human). The program calculated that the insulin receptor family proteins are clearly divided into two classes: one class with an optimal working pH in acidic media (virtually all insulin receptor and insulin-like growth factor receptor orthologs, except for the IGF-IR ortholog from *Xenopus laevis*); and the second class with the optimal working pH in alkaline media (all IRR orthologs). For example, human IR was classified as an “acid-dependent” protein with a probability of 0.95 and as an “alkali-dependent” protein, with a probability of 0.05. However, for human IRR, we found that the probability of being “acid-dependent” was 0.25, and 0.75 for being an “alkali-dependent” protein. Indeed, within the insulin receptor family, the alkali-sensing property is a unique feature of IRR.

This simple test encouraged us to perform a full analysis of all human receptor tyrosine kinases’ ectodomains, and we found 17 receptors to be potential alkali-sensing receptors (Table S1). Next, we determined the probability of pH sensitivity in the orthologs of these receptors from various mammalian species and found that only 9 receptors were predicted as “alkali-dependent” proteins (Table 1). One of these, the ErbB2 drew our attention because it was an orphan receptor with a reported inability to bind peptide ligands [2].

**Figure 1:**
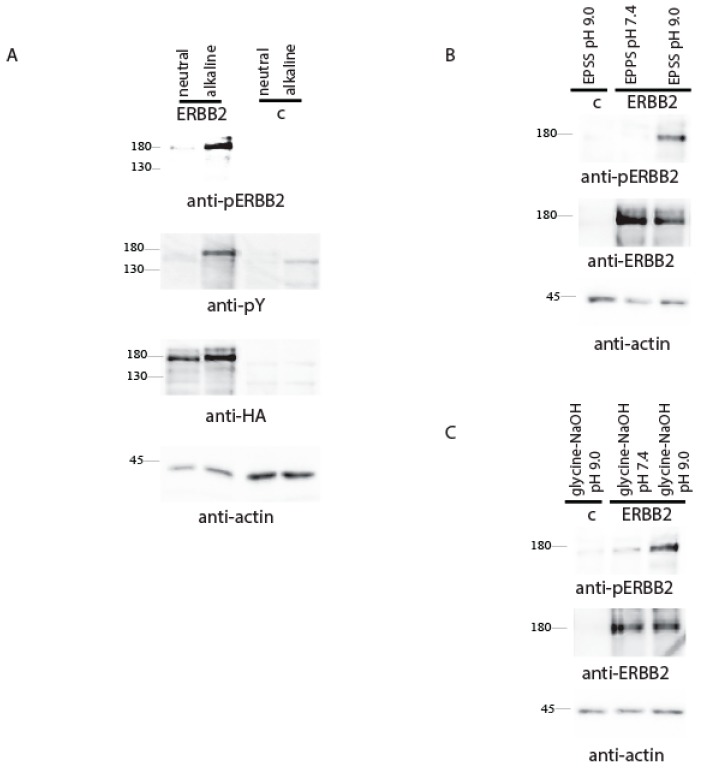
(**A**) HEK293 cells were transfected with a HA-tagged *ErbB2*-coding construct. At 36 h after transfection, the cells were incubated in PBS at pH 7.4 or 9.0 adjusted by 60 mM Tris-HCl for 10 min. Then, the cells were lysed and analyzed by Western blotting with the indicated antibodies. Control cells are untransfected cells. (**B**) HEK293 cells were transfected with HA-tagged *ErbB2*-coding construct and then incubated in PBS solution at pH 7.4 or 9.0 adjusted by 60 mM EPPS buffer with the same pH for 10 min. The cells were lysed and blotted with the indicated antibodies. (**C**) HEK293 cells were transfected with HA-tagged *ErbB2*-coding construct and then incubated in PBS solution at pH 7.4 or 9.0 adjusted by 60 mM glycine–NaOH buffer with the same pH for 10 min. The cells were lysed and blotted with the indicated antibodies.

To test whether ErbB2 can be stimulated by the alkali media, we transfected HEK293 cells with human cDNA of *ErbB2* with HA-tag at the receptor C-terminus followed by incubation in Tris-buffered physiological saline solutions at pH 7.4 or 9.0, and found a robust ErbB2 autophosphorylation response upon the alkali treatment (Figure 1A). Then, we examined the activation of ErbB2 receptor by alkaline pH in two additional different buffer systems (Figure 1B) and revealed that the pH-sensing property of ErbB2 was buffer-independent.

**Figure 2:**
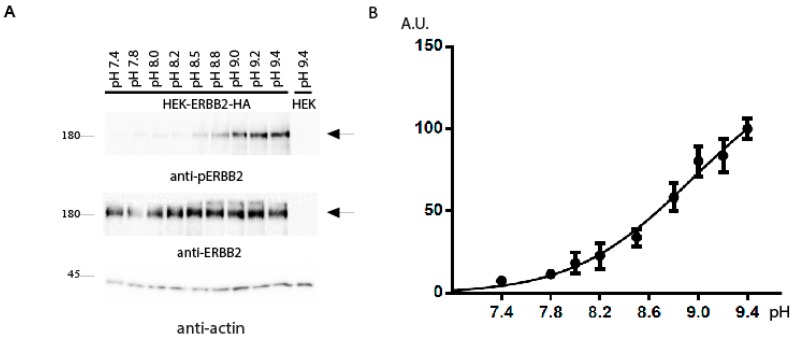
(**A**) pH-dependence of ErbB2 activation by alkaline media. HEK293 cells were transfected with the HA-tagged *ErbB2* coding construct. At 36 h after transfection, the cells were incubated in PBS with the indicated pH adjusted by Tris-HCl, lysed and blotted with anti-phosphoErbB2 (anti-pErbB2), anti-ErbB2, and anti-actin antibodies. (**B**) Quantitative analysis of four independent experiments. Phosphorylation signals from Western blots were quantified and normalized according to the anti-ErbB2 signals. Normalized signals were plotted vs. pH of the tested solutions. Values are means ± SE (*n* = 4).

To analyze the pH dependence of the ErbB2 response in detail, HEK293 cells transfected with *ErbB2* were incubated with a set of Tris-buffered physiological saline solutions with pH varying from 7.4 to 9.4 in small increments. Cell lysates were directly analyzed by Western blotting with anti-phosphoErbB2 and anti-ErbB2 antibodies (Figure 2A). Western blot analysis revealed a dose-dependent activation of ErbB2 by alkali. The ratio of integral density of the phosphorylated receptor (pErbB2/ErbB2 signal) was plotted versus pH (Figure 2B), and indicated statistically significant autophosphorylation of ErbB2 at pH above 8.2.

**Figure 3:**
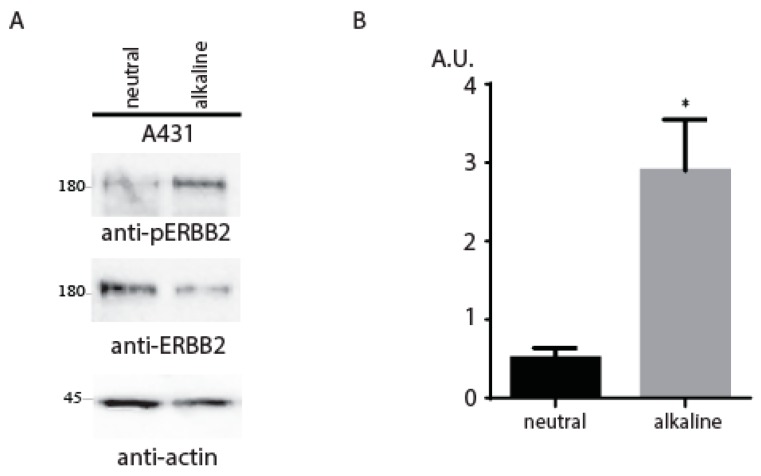
(**A**) A431 were starved and incubated in F-12 medium with the indicated pH adjusted by 60 mM Tris-HCl buffer for 10 min. The cells were lysed and lysates were blotted with anti-phosphoErbB2, anti-ErbB2, and anti-actin antibodies. (**B**) Quantitative analysis of four independent experiments. Phosphorylation signals from Western blots were quantified and normalized according to the anti-ErbB2 signals. The normalized signals were plotted. Values are means ± SE (*n* = 4). Asterisks indicate *p* < 0.05.

Finally, we tested the pH-sensing property of the endogenously present ErbB2 receptor in A431 cell lines with a physiologically relevant level of ErbB2 expression. These cells were incubated in Tris-buffered physiological saline solutions at pH 7.4 or 8.6. The cells were further lysed and blotted with anti-ErbB2 antibody or anti-phosphoErbB2 antibody (Figure 3A). The quantitative analysis of ErbB2 receptor phosphorylation showed statistically significant ErbB2 activation in A431 cells by the alkali medium (Figure 3B).

The research interest towards ErbB2 is primarily based on its role in oncogenesis, yet, the physiological function of ErbB2 in normal cells or tissues remains poorly understood. This fact is primarily associated with the lack of identified specific ligands or agonists of ErbB2. However, it was shown that due to the formation of heterodimeric complexes with EGFR or ErbB3 on the cell membrane, it can be stimulated with neuregulin. Thus, the current model of ErbB2 activation includes two mechanisms: one is the oncogenic way, representing the formation of ErbB2 homodimers due to non-physiologically high concentrations of overexpresssed ErbB2 or oncogenic mutations in the *ErbB2* gene; and the other one is a heterodimeric complex formation with another member of the ErbB receptor family. Here, on the basis of the AcalPred program prediction, we report the unexpected finding that ErbB2 can be activated by extracellular alkaline media. This hypothesis is supported by transfection experiments with *ErbB2* cDNA in HEK293 cells and by the analysis of the endogenous ErbB2 phosphorylation in A431 cells.

Our previous studies of IRR demonstrated that it functions as an alkaline pH sensor. Also, by the analysis of IRR knockout mice, its role in the regulation of the acid–base balance by kidneys was revealed. The finding of a similar property in ErbB2 raises the possibility that this receptor may also participate in systemic functions that involve bases or acids. Most research data have focused on the role of ErbB2 in oncogenesis and tumor progression, and there are only few reports about the physiological role of ErbB2 in normal tissues [11].

In healthy animals, ErbB2 is preferentially expressed in salivary glands, colon mucosa, gastric mucosa, and the endocrine and exocrine parts of the pancreas [12,13]. In these tissues, alkaline pH can be observed in the extracellular medium [14,15]. The pH range in saliva may vary from 6 to 8.6, depending on the state of the organism [16,17]. The ability to maintain an alkaline pH in saliva is important because saliva in the oral cavity plays an antibacterial role and swallowing saliva can also compensate for acidic pH in the stomach, especially in cases of reflux disease [18]. Several reports show that the pH of colon or gastric mucosa can reach 8–8.2 and, in particular, the alkaline contents of gastric mucosa serve to protect the stomach from the acidic contents [19,20]. One of the key functions of the pancreas is to secrete mildly alkaline (pH 8–8.5) juice to the intestine to facilitate digestion and absorption [21]. Thus, the anatomy of ErbB2 distribution supports its potential role as a pH-sensing receptor.

Knockout of the *ErbB2* gene in mice results in an embryonic (E11) lethal phenotype due to defects in cardiac ventricular myocyte differentiation [22], as well as development of cranial neural crest-derived sensory ganglia that are markedly affected in *ErbB2* knockout mice [22,23]. Due to the lack of literature data on the role of extracellular alkali in the regulation of mammalian development, it is not yet clear how the pH-sensing property of ErbB2 can contribute to the described *ErbB2* knockout phenotypes. Yet, it should be noted that ErbB2 shows strong expression in dorsal root ganglia during embryogenesis. A similar pattern of expression is characteristic for alkali sensor IRR and acid sensors ASICS, suggesting that these neurons may be regulated by extracellular pH changes [24,25].

## 3. Materials and Methods

### 3.1. Cell Lines and Treatments

A431 and HEK293 cell lines were cultured in DMEM supplemented with 10% fetal bovine serum (Hyclone), 1% penicillin/streptomycin, and 2 mM l-glutamine. The HEK293 cells with a density of about 50% were transfected with cDNA of human *ErbB2* receptor with HA tag on the C-terminal end of the receptor using unifectin-56 (Unifect Group, Moscow, Russia), according to manufacturer’s protocols. At 36 h after transfection, cells were washed with serum-free F-12 and incubated for 3 h in serum-free F-12 containing 1% penicillin/streptomycin in a CO_2_ incubator. The cells were further incubated in PBS with 60 mM Tris-HCl with different pH values at room temperature and lysed in the SDS-PAGE sample buffer (75 mM Tris-HCl pH 6.8, 1.5% SDS, 150 mM β-mercaptoethanol, and 15% glycerol).

To analyze the pH dependence of ErbB2 activation, transfected cells were incubated with a set of Tris-buffered physiological saline solutions at various pH ranging from 7.4 to 9.4 in small increments. The transfected cells were lysed with sample buffer, separated by electrophoresis, and analyzed by Western blotting with anti-pErbB2, anti-ErbB2, and anti-actin antibodies. The blots were visualized by chemiluminescence that was captured using a Fusion Solo system. The ratio of integral density of the phosphorylated receptor (pErbB2 signal) to the total receptor (ErbB2 antibody signal) was plotted versus pH.

A431 cells in a confluent monolayer were starved in serum-free F-12 medium for 3 h to decrease basal protein phosphorylation. To activate the receptor tyrosine kinase ErbB2, cells were incubated in serum-free F-12 medium adjusted with 25 mM Tris-HCl at the indicated pH for 15 min at 37 °C, then the cells were lysed with SDS loading buffer and processed by Western blotting.

### 3.2. Antibodies and Immunoblotting

The cell lysates were separated by electrophoresis in 8% SDS-PAGE gel followed by blotting onto ECL-grade nitrocellulose (Amersham, GE Healthcare, Chicago, IL, USA) as described in [26]. The blots were probed with monoclonal anti-phosphotyrosine antibody 4G10 (Millipore, Merck KGaA, Darmstadt, Germany) and with rabbit anti-phosphoErbB2 (Cell Signaling Technology, Leiden, Netherlands) and rabbit anti-ErbB2 (Cell Signaling Technology) antibodies. Blots were blocked overnight in 5% non-fat milk or 1% BSA (for phosphoprotein detection) in a TBST buffer (10 mM Tris-HCl, pH 7.8, 150 mM NaCl, and 0.1% Tween 20) and then incubated with primary antibodies. After incubation with horseradish peroxidase-conjugated anti-rabbit or anti-mouse secondary antibodies (Jackson ImmunoResearch, West Grove, PA, USA), immunoreactive bands were visualized by enhanced chemiluminescence (Pierce, New Brighton, MN, USA). For the quantitative analysis of Western blots, we used the Fusion Solo system (Vilber Lourmat, France). The captured images were manually selected in rectangles and further analyzed by densitometry with Fusion software (Vilber Lourmat), the background was subtracted by selecting non-stained blot areas. Final calculations were made using GraphPad 6.0.1 software (GraphPad Software, La Jolla, CA, USA).

## Figures and Tables

**Table 1:** Sequence analysis of potential “alkali-sensing” receptor tyrosine kinases’ ectodomains from various mammalian species using the AcalPred program. Proteins with a predicted probability of alkaline sensitivity greater than 0.5 are shown in red.

Receptor	Xenopus	Mouse	Rat	Dog	Chicken	Human
ERBB2	0.83	0.806	0.838	0.773	0.633	0.696
ERBB3	0.622	0.853	0.746		0.807	0.595
INSRR	0.573	0.798	0.912	0.839	0.546	0.726
PDGFRB	0.819	0.111	0.141	0.786	0.641	0.874
FLT3	0.829	0.834	0.767	0.844	0.944	0.947
VEGFR1		0.909		0.905	0.924	0.976
CCK4	0.975	0.892		0.923	0.989	0.803
MST1R	0.808	0.819	0.847	0.877	0.464	0.634
EPHB4	0.888	0.905	0.916	0.594		0.685
TIE1	0.564	0.358	0.464	0.608	0.33	0.787
TIE2	0.978	0.896	0.905	0.854	0.539	0.888
DDR2	0.612	0.657	0.688	0.367	0.965	0.646
RET	0.258	0.618	0.381	0.739	0.173	0.667
ROR1	0.953	0.856	0.969	0.227	0.771	0.939
ROR2	0.232	0.679	0.554	0.699	0.9	0.793
MUSK	0.693	0.99	0.984	0.947	0.908	0.972

## Supplementary Materials

Supplementary materials can be found at https://www.mdpi.com/1422-0067/20/6/1515/s1.
