# Supplementary material for: Autophosphorylation of Orphan Receptor ERBB2 Can Be Induced by Extracellular Treatment with Mildly Alkaline Media
Source: Int J Mol Sci. 2019 Mar 26;20(6):1515. doi: 10.3390/ijms20061515 (PMC6470685; doi:10.3390/ijms20061515)
Supplement: Supplementary file 1 [file ijms-20-01515-s001.pdf]

Supplement Table 1. Sequence analysis of human tyrosine kinase receptor's ectodomains from using the AcalPred program. Proteins with a predicted probability of alkaline sensitivity greater than 0.5 are shown as "red". Classification according to Aude I. Ségaliny et. al., "Receptor tyrosine kinases: Characterisation, mechanism of action and therapeutic interests for bone cancers", Journal of Bone Oncology 2015, PMID: 26579483.

RTK class I (EGF receptor family)

	**Name**	**Result: acidic/alkaline**
1.	1956 EGFR, ERBB, ERBB1, HER1, PIG61, mENA; epidermal growth factor receptor (EC:2.7.10.1)	acidic enzyme: 0.884038/0.115962
2.	2064 ERBB2, CD340, HER-2, HER-2/neu, HER2, MLN_19, NEU, NGL, TKR1; v-erb-b2 erythroblastic leukemia viral oncogene homolog 2	alkaline enzyme: 0.304008/0.695992
3.	2065 ERBB3, ErbB-3, HER3, LCCS2, MDA-BF-1, MGC88033, c-erbB-3, c-erbB3, erbB3-S, p180-ErbB3, p45-sErbB3, p85-sErbB3; v-erb-b2	alkaline enzyme: 0.404655/0.595345
4.	2066 ERBB4, HER4, MGC138404, p180erbB4; v-erb-a erythroblastic leukemia viral oncogene homolog 4 (avian) (EC:2.7.10.1)	acidic enzyme: 0.716044/0.283956

RTK class II (Insulin receptor family)

	**Name**	**Result: acidic/alkaline**
1.	3643 INSR, CD220, HHF5; insulin receptor (EC:2.7.10.1)	acidic enzyme: 0.924851/0.075149
2.	3645 INSRR, IRR; insulin receptor-related receptor (EC:2.7.10.1)	alkaline enzyme: 0.273692/0.726308
3.	3480 IGF1R, CD221, IGFIR, IGFR, JTK13, MGC142170, MGC142172, MGC18216; insulin-like growth factor 1 receptor (EC:2.7.10.1)	acidic enzyme: 0.911275/0.088725

RTK class III (PDGF receptor family)

	**Name**	**Result: acidic/alkaline**
1.	5156 PDGFRA, CD140A, MGC74795, PDGFR2, RHEPDGFRA; platelet-derived growth factor receptor, alpha polypeptide (EC:2.7.10.1)	acidic enzyme: 0.881971/0.118029
2.	5159 PDGFRB, CD140B, JTK12, PDGFR, PDGFR1; platelet-derived growth factor receptor, beta polypeptide (EC:2.7.10.1)	alkaline enzyme: 0.126319/0.873681
3.	1436 CSF1R, C-FMS, CD115, CSFR, FIM2, FMS; colony stimulating factor 1 receptor (EC:2.7.10.1)	acidic enzyme: 0.647589/0.352411
4.	3815 KIT, C-Kit, CD117, PBT, SCFR; v-kit Hardy-Zuckerman 4 feline sarcoma viral oncogene homolog (EC:2.7.10.1)	acidic enzyme: 0.873693/0.126307
5.	2322 FLT3, CD135, FLK2, STK1; fms-related tyrosine kinase 3	alkaline enzyme:

	(EC:2.7.10.1)	0.053412/0,946588

RTK class IV (VEGF receptor family)

	**Name**	**Result: acidic/alkaline**
1.	2321 VEGFR1, FLT1, FLT; fms-related tyrosine kinase 1 (vascular endothelial growth factor/vascular permeability factor receptor)	alkaline enzyme: 0.024028/0.975972
2.	2324 VEGFR3, FLT4, FLT41, LMPH1A, PCL; fms-related tyrosine kinase 4 (EC:2.7.10.1)	acidic enzyme: 0.807917/0.192083
3.	3791 VEGFR2, KDR, CD309, FLK1, VEGFR; kinase insert domain receptor (a type III receptor tyrosine kinase) (EC:2.7.10.1)	acidic enzyme: 0.523830/0.476170

RTK class V (FGF receptor family)

	**Name**	**Result: acidic/alkaline**
1.	2260 FGFR1, BFGFR, CD331, CEK, FGFBR, FLG, FLJ99988, FLT2, HBGFR, KAL2, N-SAM, OGD; fibroblast growth factor receptor 1 (EC:2.7.10.1)	acidic enzyme: 0.970732/0.029268
2.	2263 FGFR2, BEK, BFR-1, CD332, CEK3, CFD1, ECT1, FLJ98662, JWS, K-SAM, KGFR, TK14, TK25; fibroblast growth factor receptor 2 (EC:2.7.10.1)	acidic enzyme: 0.641411/0.358589
3.	2261 FGFR3, ACH, CD333, CEK2, HSFGFR3EX, JTK4; fibroblast growth factor receptor 3 (EC:2.7.10.1)	acidic enzyme: 0.877988/0.122012
4.	2264 FGFR4, CD334, JTK2, MGC20292, TKF; fibroblast growth factor receptor 4 (EC:2.7.10.1)	acidic enzyme: 0.884513/0.115487

RTK class VI (CCK4 receptor family)

	**Name**	**Result: acidic/alkaline**
1.	5754 CCK4, PTK7; PTK7 protein tyrosine kinase 7 (EC:2.7.10.1)	alkaline enzyme: 0.197042/0.802958

RTK class VII (TRK receptor family)

	**Name**	**Result: acidic/alkaline**
1.	4914 NTRK1, DKFZp781I14186, MTC, TRK, TRK1, TRKA, Trk-A, p140-TrkA; neurotrophic tyrosine kinase, receptor, type 1 (EC:2.7.10.1)	acidic enzyme: 0.760652/0.239348
2.	4915 NTRK2, GP145-TrkB, TRKB; neurotrophic tyrosine kinase, receptor,	acidic enzyme:

	type 2 (EC:2.7.10.1)	0.992919/0.007081
3.	4916 NTRK3, TRKC, gp145(trkC); neurotrophic tyrosine kinase, receptor, type 3 (EC:2.7.10.1)	acidic enzyme: 0.871313/0.128687

RTK class VIII (HGF receptor family)

	**Name**	**Result: acidic/alkaline**
1.	4233 MET, AUTS9, HGFR, RCCP2, c-Met; met proto-oncogene (hepatocyte growth factor receptor) (EC:2.7.10.1)	acidic enzyme: 0.639559/0.360441
2.	4486 MST1R, CD136, CDw136, PTK8, RON; macrophage stimulating 1 receptor (c-met-related tyrosine kinase) (EC:2.7.10.1)	alkaline enzyme: 0.365520/0.634480

RTK class IX (EPH receptor family)

	**Name**	**Result: acidic/alkaline**
1.	2041 EPHA1, EPH, EPHT, EPHT1, MGC163163; EPH receptor A1 (EC:2.7.10.1)	acidic enzyme: 0.888125/0.111875
2.	1969 EPHA2, ARCC2, ECK; EPH receptor A2 (EC:2.7.10.1)	acidic enzyme: 0.865633/0.134367
3.	2042 EPHA3, EK4, ETK, ETK1, HEK, HEK4, TYRO4; EPH receptor A3 (EC:2.7.10.1)	acidic enzyme: 0.970543/0.029457
4.	2043 EPHA4, HEK8, SEK, TYRO1; EPH receptor A4 (EC:2.7.10.1)	acidic enzyme: 0.988390/0.011610
5.	2044 EPHA5, CEK7, EHK1, HEK7, TYRO4; EPH receptor A5 (EC:2.7.10.1)	acidic enzyme: 0.748708/0.251292
6.	285220 EPHA6, DKFZp434C1418, EPA6, FLJ35246, PRO57066; EPH receptor A6 (EC:2.7.10.1)	no data
7.	2045 EPHA7, EHK3, HEK11; EPH receptor A7 (EC:2.7.10.1)	acidic enzyme: 0.687533/0.312467
8.	2046 EPHA8, EEK, EK3, HEK3, KIAA1459; EPH receptor A8 (EC:2.7.10.1)	alkaline enzyme: 0.450233/0.549767
9.	2047 EPHB1, ELK, EPHT2, FLJ37986, Hek6, NET; EPH receptor B1 (EC:2.7.10.1)	acidic enzyme: 0.773047/0.226953
10.	2048 EPHB2, CAPB, DRT, EK5, EPHT3, ERK, Hek5, MGC87492, PCBC, Tyro5; EPH receptor B2 (EC:2.7.10.1)	acidic enzyme: 0.698489/0.301511
11.	2049 EPHB3, ETK2, HEK2, TYRO6; EPH receptor B3 (EC:2.7.10.1)	acidic enzyme: 0.851291/0.148709
12.	2050 EPHB4, HTK, MYK1, TYRO11; EPH receptor B4 (EC:2.7.10.1)	alkaline enzyme: 0.314595/0.685405
13.	2051 EPHB6, HEP, MGC129910, MGC129911; EPH receptor B6	acidic enzyme:

	(EC:2.7.10.1)	0.773149/0.226851

RTK class X (AXL receptor family)

	Name	Result: acidic/alkaline
1.	558 AXL, JTK11, UFO; AXL receptor tyrosine kinase (EC:2.7.10.1)	acidic enzyme: 0.640161/0.359839
2.	7301 TYRO3, BYK, Dtk, FLJ16467, RSE, Sky, Tif; TYRO3 protein tyrosine kinase (EC:2.7.10.1)	acidic enzyme: 0.899500/0.100500
3.	10461 MERTK, MER, MGC133349, RP38, c-mer; c-mer proto-oncogene tyrosine kinase (EC:2.7.10.1)	acidic enzyme: 0.835043/0.164957

RTK class XI (TIE receptor family)

	Name	Result: acidic/alkaline
1.	7075 TIE1, JTK14, TIE; tyrosine kinase with immunoglobulin-like and EGF-like domains 1 (EC:2.7.10.1)	alkaline enzyme: 0.212808/0.787192
2.	7010 TEK, CD202B, TIE-2, TIE2, VMCM, VMCM1; TEK tyrosine kinase, endothelial (EC:2.7.10.1)	alkaline enzyme: 0.111874/0.888126

RTK class XII (RYK receptor family)

	Name	Result: acidic/alkaline
1.	6259 RYK, D3S3195, JTK5, JTK5A, RYK1; RYK receptor-like tyrosine kinase (EC:2.7.10.1)	acidic enzyme: 0.695962/0.304038

RTK class XIII (DDR receptor family)

	Name	Result: acidic/alkaline
1.	780 DDR1, CAK, CD167, DDR, EDDR1, HGK2, MCK10, NEP, NTRK4, PTK3, PTK3A, RTK6, TRKE; discoidin domain receptor tyrosine kinase 1	acidic enzyme: 0.964024/0.035976
2.	4921 DDR2, MIG20a, NTRKR3, TKT, TYRO10; discoidin domain receptor tyrosine kinase 2 (EC:2.7.10.1)	alkaline enzyme: 0.354485/0.645515

RTK class XIV (RET receptor family)

	Name	Result: acidic/alkaline
1.	5979 RET, CDHF12, CDHR16, HSCR1, MEN2A, MEN2B, MTC1, PTC, RET-ELE1, RET51; ret proto-oncogene (EC:2.7.10.1)	alkaline enzyme: 0.333400/0.666600

RTK class XV (ROS receptor family)

	Name	Result: acidic/alkaline
4.	6098 ROS1, MCF3, ROS, c-ros-1; c-ros oncogene 1, receptor tyrosine kinase (EC:2.7.10.1)	acidic enzyme: 0.859824/0.140176

RTK class XVI (LTK receptor family)

	Name	Result: acidic/alkaline
1.	4058 LTK, TYK1; leukocyte receptor tyrosine kinase (EC:2.7.10.1)	acidic enzyme: 0.560120/0.439880
2.	238 ALK, CD246, NBLST3, TFG/ALK; anaplastic lymphoma receptor tyrosine kinase (EC:2.7.10.1)	acidic enzyme: 0.515580/0.484420

RTK class XVII (ROR receptor family)

	Name	Result: acidic/alkaline
1.	4919 ROR1, MGC99659, NTRKR1, dJ537F10.1; receptor tyrosine kinase-like orphan receptor 1 (EC:2.7.10.1)	alkaline enzyme: 0.060833/0.939167
2.	4920 ROR2, BDB, BDB1, MGC163394, NTRKR2; receptor tyrosine kinase-like orphan receptor 2 (EC:2.7.10.1)	alkaline enzyme: 0.206668/0.793332

RTK class XVIII (MuSK receptor family)

	Name	Result: acidic/alkaline
1.	4593 MUSK, MGC126323, MGC126324; muscle, skeletal, receptor tyrosine kinase (EC:2.7.10.1)	alkaline enzyme: 0.028467/0.97153
